# Association of whole genome sequencing variants with time to Alzheimer's disease

**DOI:** 10.1002/alz70855_107062

**Published:** 2025-12-25

**Authors:** Mohsen Sharifi Tabar, Dongyu Wang, Gina M. Peloso, Josh C. Bis, Bruce M. Psaty, Anita L. DeStefano, Habil Zare, Sudha Seshadri, Xueqiu Jian

**Affiliations:** ^1^ The University of Texas Health Science Center at San Antonio, San Antonio, TX, USA; ^2^ Boston University, Boston, MA, USA; ^3^ University of Washington, Seattle, WA, USA

## Abstract

**Background:**

Alzheimer's disease (AD) risk is influenced by complex genetic factors. Genome‐wide association studies (GWAS) have identified several AD‐associated loci, primarily in case‐control samples. Despite heritable factors are known to play an important role in the timing of AD onset, GWAS specifically focused on time‐to‐AD remain limited.

**Method:**

We utilized data from the Alzheimer's Disease Sequencing Project (ADSP) and two major cohorts in the Trans‐Omics for Precision Medicine (TOPMed) program, encompassing over 14,000 cognitively normal individuals at baseline who have both time‐to‐AD and whole genome sequencing (WGS) data. Our current sample included approximately 11,000 non‐Hispanic whites (NHW), 1,700 blacks, and 1,400 Hispanics. Ancestry‐specific Cox proportional hazards model was applied in each dataset separately, adjusting for sex, baseline age, study/site, genetic principal components for population structure, and kinship matrix for familial relatedness (in TOPMed only).

**Result:**

Our initial analysis, focusing on NHW, revealed an AD incidence rate of 0.0344 per person‐year in ADSP and 0.0138 in TOPMed. Analysis of variants with minor allele frequency (MAF) >1% showed the APOE region as the strongest association in both datasets. Additionally, several other variants reached genome‐wide significance (*p* <5e‐8), including those mapped to ROR1 (1p31.3), ROR2 (9q22.31), ADAMTS8 (11q24.3), and SHISA6 (17p12). ROR1 and ROR2 both encode a subfamily of cell surface receptors and are involved in neuronal development, synaptic function, Wnt signaling, and inflammation, suggesting their potential contribution to AD pathogenesis. ADAMTS8 encodes a proteolytic enzyme likely involved in neuroplasticity and degradation, while SHISA6 is implicated in hippocampal synaptic transmission and has been linked to delayed cognitive impairment.

**Conclusion:**

Our preliminary findings highlight the critical role of APOE in the timing of AD onset, while also identifying potential novel loci associated with the disease. Except for APOE, there was limited overlap between our Cox GWAS results and those from case‐control GWAS, suggesting distinct genetic factors influencing AD risk over time. Meta‐analysis of the results from the two datasets, along with assessment of rarer variants, are underway. We will also perform multi‐ancestral analysis and seek to expand the sample size for black and Hispanic populations.

